# Amphistome infections in domestic and wild ruminants in East and Southern Africa: A review

**DOI:** 10.4102/ojvr.v85i1.1584

**Published:** 2018-10-18

**Authors:** Davies M. Pfukenyi, Samson Mukaratirwa

**Affiliations:** 1 Faculty of Veterinary Science, University of Zimbabwe, Zimbabwe; 2 School of Life Sciences, University of KwaZulu-Natal, South Africa

## Abstract

In this article, the main amphistome species infecting domestic and wild ruminants in East and Southern Africa, their snail intermediate hosts and epidemiological features are reviewed and discussed. Twenty-six amphistome species belonging to nine genera from three families occur in domestic and wild ruminants in the region under review and over 70% of them belong to the genera *Calicophoron, Carmyerius* and *Cotylophoron*. Of the amphistome species, 76.9% are shared between domestic and wild ruminant hosts – an important observation when considering the different options for control. Seven freshwater snail species belonging to four genera from two families act as intermediate hosts of the identified amphistome species, with the genus *Bulinus* contributing 57% of the snail species. Some of the snails are intermediate hosts of amphistome species belonging to the same genus or to different genera; a phenomenon not yet fully elucidated as some snails are reported to be naturally infected with amphistome cercariae of unidentified species. Only nine (34.6%, 9/26) of the amphistome species have known snail intermediate hosts, while most (65.4%, 17/26) have unknown hosts. Species of intermediate hosts and the potential of the flukes to infect these hosts, the biological potential of the snail hosts, the definitive hosts management systems and their grazing habits are considered to be the main factors influencing the epidemiology of amphistomosis. Based on the epidemiological features of amphistome infections, various practical control options are discussed. Further research is necessary to determine amphistome–snail associations, develop diagnostic tests that can detect prepatent infections in the definitive host, determine the burden and economic importance of amphistomosis in domestic and wild ruminants and the efficacy of different anthelmintics in the treatment of patent infections.

## Introduction

Amphistomosis is a disease of domestic and wild ruminants caused by digenetic trematodes of the superfamily Paramphistomoidea Fischoeder, 1901 (Lotfy et al. 2010). The superfamily has a cosmopolitan distribution and is composed of hundreds of species belonging to 12 families (Jones 2005). Given their ubiquity and their abundance within hosts, it seems likely that the importance of these flukes is underestimated globally (Lotfy et al. 2010). Various species of the different paramphistomoid families, especially members of Paramphistomidae and Gastrothylacidae, cause amphistomosis among ruminants. The disease is caused by a severe infection with immature flukes in the small intestines of immunologically incompetent hosts. The amphistomes are responsible for lower nutrition conversion and result in a loss of weight and/or a decrease in milk production, causing great economic losses (Horak 1971). However, most reports on the disease do not quote the responsible amphistome species as they are difficult to identify from a systematic point of view (Horak 1971). *Calicophoron microbothrium* is probably the biggest cause of this disease in Africa (Dinnik 1964a). Knowledge of the different amphistome species infecting domestic and wild ruminants facilitates a better understanding of the amphistome–host associations and the epidemiology of the disease.

A wide range of gastropods belonging to the genera *Bulinus* Müller 1781, *Biomphalaria* Preston 1910, *Ceratophallus* Brown and Mandahl-Barth 1973 and *Galba* Müller 1774 act as the intermediate hosts of amphistomes in Africa (Dinnik 1961, 1965; Dinnik & Dinnik 1954; Southgate et al. 1989; Wright, Southgate & Howard 1979). The prevalence of snail-borne diseases such as amphistomosis is influenced by both the abundance of infected definitive hosts and the abundance and efficiency of the snail intermediate hosts. Hence, the epidemiology and seasonal patterns of infection with amphistomes is determined to a large extent by the availability of the snail intermediate hosts and the grazing habits of the definitive hosts (Horak 1971; Rolfe et al. 1991). Information on the snail hosts of different amphistome species is essential as knowledge of the amphistome–snail associations has an influence on amphistomosis epidemiology and control.

In this review, to avoid confusion, genera of parasites and snail hosts have been abbreviated using the first three letters of the genus name and these include: for amphistomes – *Bilatorchis* (Bil.), *Calicophoron* (Cal.), *Carmyerius* (Car.), *Choerocotyloides* (Cho.), *Cotylophoron* (Cot.), *Gastrothylax* (Gas.), *Gigantocotyle* (Gig.), *Orthocoelium* (Ort.) and *Stephanopharynx* (Ste.) and for snail hosts – *Biomphalaria* (Bio.), *Bulinus* (Bul.) and *Ceratophallus* (Cer.). The authorities of the digenean families and species and that of the snail species referred to in this review can be found in Table 1.

**TABLE 1:** Checklist of amphistome species and their ruminant and snail intermediate hosts reported in east and southern African countries.

Family	Species	Country reported	Domestic ruminant hosts	Wild ruminant hosts	Intermediate snail hosts	References
Paramphistomidae Fischoeder 1901	*Bilatorchis papillogenitalis* Eduardo 1980	Zambia	–	Blue wildebeest (*Connochaetes taurinus* Burchell 1823) and Kafue lechwe (*Kobus leche* Gray 1850)	Not yet known	Eduardo 1980; Eduardo 1986;
	*Calicophoron bothriophoron* (Braun 1892) Eduardo 1983	Kenya, South Africa and Tanzania	Cattle (*Bos primigenius* Bojanus 1827), goats (*Capra hircus* Linnaeus 1758) and sheep (*Ovis aries* Linnaeus 1758)	Buffalo (*Syncerus caffer* Sparrman 1779) and Waterbuck (*Kobus ellipsiprymnus* Ogilby 1833)	Not yet known	Dinnik 1964a; Sey & Graber 1979a; Eduardo 1983
	*Calicophoron calicophorum* (Fischoeder 1901) Näsmark 1937	Angola, Kenya, Mozambique, South Africa, Zambia and Zimbabwe	Cattle, goats and sheep	Buffalo, Blue wildebeest, Bushbuck (*Tragelaphus scriptus* Pallas 1766), Eland (*Taurotragus oryx* Pallas 1766), Gemsbok (*Oryx gazella* Linnaeus 1758), Kudu (*Tragelaphus strepsiceros* Pallas 1766), Hartebeest (*Alcelaphus buselaphus* Pallas 1766), Impala (*Aepyceros melampus* Lichtenstein 1812), Kafue lechwe, Nyala (*Tragelaphus angasii* Angas 1849), Roan antelope (*Hippotragus equinus* Geoffroy Saint-Hilaire 1803), Sable antelope (*Hippotragus niger* Harris 1838), Waterbuck	*Bulinus tropicus* Krauss 1848	6, Porter 1921; 7, Porter 1938; 8 Swart 1954; 9, Caeiro 1961; 10, Ortlepp 1961; 11, Von Roth & Dalchow 1967; 12; Cruz e Silva 1971; 13; Anderson 1983; 14, Jooste 1987; 15, Jooste 1989; 16, Dube et al. 2002; 17, Dube et al. 2004; 18, Kock et al. 2002; 19, Dube & Tizauone 2014; 20, Sibula et al. 2014;
	*Calicophoron clavula* (Näsmark 1937) Eduardo 1983	Kenya, Tanzania, Uganda and Zimbabwe	Cattle, goats and sheep	Buffalo, Hartebeest, Impala and Sable antelope	Not yet known	Eduardo 1983;Jooste 1989; Dube et al. 2002; Dube et al. 2004; Dube & Tizauone 2014; Eduardo 1987;Madzingira et al. 2002; Dube et al. 2010; Laidemitt et al. 2017
	*Calicophoron daubneyi* (Dinnik 1962) Eduardo 1983	Kenya	Cattle	-	*Galba truncatula*Müller 1774	Eduardo 1983; Eduardo 1987; Dinnik 1962
Paramphistomidae	*Calicophoron microbothrium* (Fischoeder 1901) Eduardo 1983	Angola, Botswana, Kenya, Lesotho, Mozambique, South Africa, Tanzania, Uganda, Zambia and Zimbabwe	Cattle, goats and sheep	Bohor reedbuck (*Redunca redunca* Pallas 1767), Buffalo, Eland, Hartebeest, Impala, Kafue lechwe, Kudu, Mountain gazelle (*Gazella gazella* Pallas 1766), Nyala, Reedbuck (*Redunca arundinum* Boddaert 1785), Roan antelope, Sable antelope, Thomson’s gazelle (*Eudorcas thomsonii* Günther 1884), Topi (*Damaliscus lunatus jimela* Matschie 1892), Tsessebe (*Damaliscus lunatus* Burchell 1824), Ugandan kob (*Kobus kob thomasi* Sclater 1896), Waterbuck	*Bulinus forskalii* Ehrenberg 1831 *Bulinus globosus* Morelet 1866 *Bulinus nasutus* von Martens 1879 *Bul. tropicus*	Dinnik 1964a; Eduardo 1983; Swart 1954; Caeiro 1961; Ortlepp 1961; Von Roth & Dalchow 1967; Cruz e Silva 1971; Jooste 1989; Dube et al. 2002; Dube et al. 2004; Kock et al. 2002; Dube & Tizauone 2014;, Eduardo 1987; Madzingira et al. 2002; Dube et al. 2010; Laidemitt et al. 2017; Dinnik & Dinnik 1954; Dinnik & Dinnik 1955; Dinnik & Dinnik 1962; Dinnik et al. 1962; Dinnik 1965; Roach & Lopes 1966; Horak 1967; Wandera 1969; Fitzsimmons 1971; Keyyu et al. 2006; Lotfy et al. 2010;Dube et al. 2015;
	*Calicophoron phillerouxi* (Dinnik 1961) Eduardo 1983	Kenya, South Africa, Tanzania, Uganda, Zambia and Zimbabwe	Cattle, goats and sheep	Bohor reedbuck, Buffalo, Kudu, Impala, Puku (*Kobus vardonii* Livingstone 1857), Reedbuck, Roan antelope, Sable antelope, Topi, Ugandan kob, Waterbuck	*Bul. forskalii*	Eduardo 1983; Von Roth & Dalchow 1967; Jooste 1987; 15, Jooste 1989; Dube et al. 2004; Eduardo 1987; Laidemitt et al. 2017; Dinnik et al. 1962; Dinnik 1961; Dinnik & Hammond 1968
	*Calicophoron raja* Näsmark 1937	Botswana, Kenya, Namibia, South Africa, Tanzania, Zambia and Zimbabwe	Cattle, goats and sheep	Blue wildebeest, Buffalo, Bushbuck, Eland, Gemsbok, Kudu, Hartebeest, Impala, Kafue lechwe, Puku, Reedbuck, Roan antelope, Sable antelope, Thomson’s gazelle, Topi, Tsessebe, Waterbuck	*Bul. globosus*	Dinnik 1964a; Eduardo 1983; Von Roth & Dalchow 1967; Jooste 1989; Dube et al. 2002; Dube et al. 2004; Dube & Tizauone 2014; Eduardo 1987; Madzingira et al. 2002; Dinnik & Dinnik 1954; Dinnik & Dinnik 1955;Dinnik & Hammond 1968; Mettrick 1962
	*Calicophoron sukari* (Dinnik 1954) Eduardo 1983	Angola, Kenya, Tanzania, Uganda, Zambia and Zimbabwe	Cattle, sheep and goats	Buffalo	*Biomphalaria pfeifferi* Krauss 1848	Dinnik 1964a; Eduardo 1983; Von Roth & Dalchow 1967; Dube et al. 2004;, Eduardo 1987;Dinnik & Dinnik; Dinnik 1965; Dinnik & Hammond; Dinnik 1954; Dinnik & Dinnik 1957; Sachs & Sachs 1968
	*Calicophoron sukumum* (Dinnik 1964) Eduardo 1983	Tanzania, Zambia and Zimbabwe	Cattle	Buffalo, Blue wildebeest, Eland, Kafue lechwe, Reedbuck, Roan antelope, Topi, Waterbuck	Not yet known	Eduardo 1983; Von Roth & Dalchow 1967; Jooste 1989; Sachs & Sachs 1968; Dinnik 1964b
	*Cotylophoron cotylophorum* (Fischoeder 1901) Stiles and Goldberger 1910	Angola, Kenya, Malawi, Mozambique, South Africa, Tanzania, Uganda, Zambia and Zimbabwe	Cattle, goats and sheep	Blue wildebeest, Bohor reedbuck, Buffalo, Bushbuck, Common duiker (*Sylvicapra grimmia* Linnaeus 1758), Eland, Kudu, Hartebeest, Kafue lechwe, Nyala, Puku, Reedbuck, Roan antelope, Sable antelope, Sitatunga (*Tragelaphus spekii* Speke 1863), Tsessebe, Topi, Ugandan kob, Waterbuck	Not yet known	Ortlepp 1961; Von Roth & Dalchow 1967; Cruz e Silva 1971; Anderson 1983; Laidemitt et al. 2017; Dinnik et al. 1962; Mettrick 1962; Le Roux 1930a; Le Roux 1930b; Le Roux 1932; Mettam 1932; Fitzsimmons 1964; Bwangamoi 1968; Eduardo 1985a;
	*Cotylophoron fuelleborni* Näsmark 1937	Kenya, Malawi, South Africa, Tanzania, Uganda and Zambia	Cattle	Buffalo and Impala	Not yet known	Anderson 1983;Dinnik et al. 1962; Eduardo 1985a
	*Cotylophoron jacksoni* Näsmark 1937	Kenya, Tanzania, Uganda, Zambia and Zimbabwe	Cattle	Kudu, Hartebeest, Impala and Sable antelope	Not yet known	Von Roth & Dalchow 1967; Jooste 1989 Madzingira et al. 2002; Keyyu et al. 2006; Eduardo 1985a
	*Cotylophoron macrosphinctris* Sey and Graber 1979b	Uganda	–	Buffalo, Hartebeest and Oribi (*Ourebia ourebi* Zimmermann 1783)	Not yet known	Eduardo 1985a; Sey & Graber 1979b; Sey 1982
	*Gigantocotyle symmeri* Näsmark 1937	Botswana, South Africa, Zambia and Zimbabwe	Cattle	Buffalo, Hartebeest, Kafue lechwe, Kudu and Roan antelope	Not yet known	Sey & Graber 1979a Dube et al. 2002; Dube & Tizauone 2014; Sibula et al. 2014; Yeh 1957; Eduardo 1984;
	*Orthocoelium scoliocoelium* (Fischoeder 1904) Yamaguti 1971	Kenya	Cattle	–	*Ceratophallus natalensis* Krauss 1848	Dinnik 1951; Dinnik 1956; Eduardo 1985b;
Gastrothylacidae Stiles and Goldberger 1910	*Carmyerius bubalis* (Innes 1912) Stunkard 1925	Zimbabwe	–	Hartebeest and Bongo (*Tragelaphus eurycerus* Ogilby 1837)	Not yet known	Sey 1983
	*Carmyerius dollfusi* Golvan Chabaud and Grétillat 1957	Botswana	Cattle	–	Not yet known	Dube et al. 2015
	*Carmyerius exoporus* Maplestone 1923	Kenya, Malawi, Tanzania and Zimbabwe	Cattle and sheep	Buffalo, Reedbuck, Roan antelope, Sitatunga, Topi and Waterbuck	*Cer.natalensis*	Dinnik 1964a; Von Roth & Dalchow 1967; Jooste 1989; Laidemitt et al. 2017; Dinnik 1965; Sey 1983; Prudhoe 1957; Dinnik & Dinnik 1960
	*Carmyerius gregarius* (Looss 1896) Stiles and Goldberger 1910	Kenya and South Africa	Cattle	Buffalo, Bushbuck and Imbabala (*Tragelaphus scriptus sylvaticus* [Pallas 1766] Sparrman 1780)	*Bulinus* species	Laidemitt et al. 2017; Sey 2000
	*Carmyerius mancupatus* (Fischoeder, 1901) Stiles and Goldberger 1910	Kenya and Tanzania	Cattle, goats and sheep	Bohor reedbuck, Buffalo, Bushbuck, Eland, Kafue lechwe, Roan antelope and Waterbuck	*Cer. natalensis*	Dinnik 1964a; Laidemitt et al. 2017;Dinnik 1965; Prudhoe 1957
	*Carmyerius parvipapillatus* Grétillat 1962	Kenya and Zambia	Cattle	Bushbuck, Sable antelope, Topi and Waterbuck	*Bul. globosus*	Dinnik 1965; Sey 1983
	*Carmyerius spatiosus* (Brandes 1898) Stiles and Goldberger 1910	Kenya, Mozambique, South Africa, Tanzania, Zambia and Zimbabwe	Cattle, goats and sheep	Bohor reedbuck, Buffalo, Bushbuck, Kafue lechwe, Hartebeest, Reedbuck, Roan antelope, Sitatunga, Topi and Waterbuck	Not yet known	Dinnik 1964a; Ortlepp 1961; Cruz e Silva 1971; Le Roux 1934; 63, Pike & Condy 1966
Gastrothylacidae	*Gastrothylax crumenifer* (Creplin 1847) Poirier 1883	South Africa and Zambia	Cattle, goats and sheep	Buffalo, Kafue lechwe and Sitatunga	Not yet known	Ortlepp 1961; Le Roux 1932
Choerocotyloididae Yamaguti 1971	*Choerocotyloides onotragi* Prudhoe, Yeh and Khalil 1964	Zambia	Cattle	Reedbuck, Red lechwe (*Kobus leche leche* Gray 1850) and Puku	Not yet known	Eduardo 1987; Pike & Condy 1966
Stephanopharyngidae Stiles and Goldberger 1910	*Stephanopharynx compactus* Fischoeder 1910	Angola, South Africa, Tanzania, Zambia and Uganda	Cattle	Blue wildebeest, Buffalo, Kafue lechwe, Reedbuck, Roan antelope and Waterbuck	Not yet known	Eduardo 1986; Dinnik 1964a; Sey & Graber 1979a; Ortlepp 1961; Dinnik 1965; Dube et al. 2015; Sey 2000

In this paper, we review the information available to date on amphistome species infecting domestic and wild ruminants in east and southern African countries, the snail intermediate hosts, as well as the epidemiology of amphistomosis and available control options.

## Amphistome species infecting ruminants in East and Southern Africa

Reported amphistome species and their respective domestic and wild ruminant hosts in east and southern African countries are shown in Table 1. Data show that the documented species belong to four families: Choerocotyloididae, Gastrothylacidae, Paramphistomidae and Stephanopharyngidae, and nine genera: one from Choerocotyloididae (*Choerocotyloides* Prudhoe, Yeh & Khalil 1964), two from Gastrothylacidae (*Carmyerius* Stiles & Goldberger 1910 and *Gastrothylax* Poirier 1883), five from Paramphistomidae (*Bilatorchis* Fischoeder, 1901, *Calicophoron* Näsmark 1937, *Cotylophoron* Stiles & Goldberger 1910, *Gigantocotyle* Näsmark 1937 and *Orthocoelium* [Stiles & Goldberger 1910] Price & McIntosh 1953) and one from Stephanopharyngidae (*Stephanopharynx* Fischoeder 1901).

Twenty-six species occur in domestic and wild ruminants in the area under review. Seventy-seven per cent of them (20/26) belong to *Calicophoron, Carmyerius* and *Cotylophoron* with the genus *Calicophoron* accounting for approximately 35% of the species, followed by *Carmyerius* (27%) and *Cotylophoron* (15%). Seventy-five per cent (9/12) of the known *Calicophoron* species and more than 40% of *Carmyerius* (43.8%, 7/16) and *Cotylophoron* (57.1%, 4/7) species occur in the area under review. However, less than 40% of *Gastrothylax* (33.3%, 1/3), *Gigantocotyle* (25%, 1/4) and *Orthocoelium* (9.1%, 1/11) known species occur in ruminants in east and southern Africa. Most of the *Calicophoron* species have a wider distribution with respect to countries where reported compared with species of the other genera. *Calicophoron microbothrium* has the widest distribution followed by *Cot. cotylophorum* and *Cal. raja. Calicophoroncalicophorum, Cal. phillerouxi, Cal. sukari, Cal. fuelleborni* and *Cal. spatiosus* also have a wider distribution compared with the rest of the other species. Seven species; *Bil. papillogenitalis, Cal. daubneyi, Car. bubalis, Car. dollfusi, Cho. Onotragi, Cot. macrosphinctris* and *Ort. scoliocoelium* had the narrowest distribution, being reported in only one country each.

The majority of the species (76.9%) are shared between domestic and wild ruminant hosts and approximately 12% of them have not been documented in domestic ruminants as yet (*Bil. papillogenitalis, Car. bubalis* and *Cot. macrosphinctris*), while another 12% (3/26) have not been reported in wild ruminants (*Cal. daubneyi, Car. dollfusi* and *Ort. scoliocoelium*). Approximately 85% (22/26) are found in domestic and 88% (23/26) in wild ruminants. All the species recorded in domestic ruminants occur in cattle with *Cal. daubneyi, Car. dollfusi* and *Ort. Scoliocoelium* documented in this ruminant host only. Sheep are hosts to 55% (12/22), while goats are hosts to 50% of the species reported in domestic ruminants. Half (11/22) of the species have been reported in all the domestic ruminants with most (63.6%, 7/11) of them being *Calicophoron* species. The range of wild ruminant hosts varies for the different species. *Cotylophoron cotylophorum* has the highest wild ruminant host range, 19 host species belonging to 10 genera followed by *Cal. raja*, 18 host species belonging to 12 genera, *Cal. microbothrium*, 17 host species belonging to 11 genera and *Car. spatiosus*, 10 host species belonging to 10 genera. *Calicophoron sukari* has the lowest wild ruminant host range, with only one wild ruminant host, that is, the buffalo. Of the wild ruminant hosts, buffaloes are hosts to 78% (18/23) of the amphistome species recorded in wild ruminants followed by the waterbuck (52.2%, 12/23), the Kafue lechwe (47.8%, 11/23), the roan antelope (47.8%, 11/23) and the hartebeest (43.4%, 10/23). The blue wildebeest, bushbuck, eland, impala, kudu and sable antelope are also hosts to more than 25% of the amphistome species documented in wild ruminant hosts.

Mixed farming systems of cattle and game, particularly antelope, have become an important agricultural activity in most east and southern African countries. In addition, there has been the creation of Transfrontier Conservation Areas (TFCAs) involving many African countries, particularly in Southern Africa, resulting in increased livestock–wildlife interface areas. Therefore, domestic and wild animals are coming into ever more intimate contact in many interface areas, particularly in rural areas at the edges of the TFCAs and in farms practising mixed cattle and game farming, thus promoting the possibility of parasite exchange. These observations are important when considering the different options for their control. For instance, Phiri et al. (2011) observed that the host range of many helminths found in the Kafue lechwe is broad and they could serve as a potentially stable source of infection to domestic animals such as goats and cattle. Hence, issues concerning livestock management and conservation may arise.

## Snail intermediate hosts

Table 1 shows the reported intermediate snail hosts of different amphistome species recorded in the study areas under review. Data show that seven snail species – *Bio. pfeifferi, Bul. forskalii, Bul. globosus, Bul. nasutus, Bul. tropicus* and *Cer. natalensis* all Planorbidae Rafinesque 1815 and *Galba truncatula* belonging to Lymnaeidae Rafinesque 1815 – are so far confirmed intermediate hosts of identified amphistome species. The genus *Bulinus* contributes 57% (4/7) of the confirmed snail intermediate hosts, while the remaining genera contribute one species each. The data also show that some snail species are intermediate hosts of amphistome species belonging to the same genus, for example, *Bul. forskalii* (*Cal. microbothrium* and *Cal. phillerouxi*), *Bul. tropicus* (*Cal. calicophorum* and *Cal. microbothrium*) and *Cer. natalensis* (*Car. exoporus* and *Car. mancupatus*) or amphistome species belonging to different genera, for example, *Bul. globosus* (*Cal. microbothrium* and *Car. parvipapillatus*) and *Cer. natalensis* (*Car. exoporus, Car. mancupatus* and *Ort. scoliocoelium*). The capacity of the various snail species to act as intermediate hosts for paramphistomoids has not been fully elucidated yet, as some of the snail hosts have been reported to be naturally infected with amphistome cercariae of unidentified species (Chingwena et al. 2002a; Dinnik 1961; Loker, Moyo & Gardner 1981; Lotfy et al. 2010; Mukaratirwa et al. 1998; Pfukenyi et al. 2005a; Wright et al. 1979). Adult amphistomes are difficult to identify using their anatomical and morphological features as they have thick robust bodies in which the internal organs are difficult to characterise (Jones 1990). As amphistomes in snail hosts are in their larval stages, species identification is made even more difficult. Hence, the technical difficulties in making precise species identifications impact greatly on the better understanding of amphistome–snail associations. However, Lotfy et al. (2010) demonstrated ITS2 as a good molecular marker for amphistome identification, which can be used to identify both adult amphistome and cercariae to species level.

The current review shows that only nine (34.6%, 9/26) amphistome species (*Cal. calicophorum, Cal. daubneyi, Cal. microbothrium, Cal. phillerouxi, Cal. sukari, Car. exoporus, Car. mancupatus, Car. parvipapillatus* and *Ort. scoliocoelium*) have known snail hosts. Except for *Cal. microbothrium*, presently with four known snail hosts (*Bul. forskalii, Bul. globosus, Bul. nasutus* and *Bul. tropicus*) in East and Southern Africa, all the other species have one known snail host each. In addition, *Bio. pfeifferi* and *Melanoides tuberculata* are known experimentally to serve as snail hosts of this parasite (Chingwena et al. 2002b). *Calicophoron microbothrium* is widely distributed in the areas under review and the wide range of its snail hosts probably supports its reported broad geographical distribution.

Data under review show that most of the known amphistome species have unknown snail hosts (65.4%, 17/26). The snail hosts of four *Calicophoron* species (*Cal.bothriophoron, Cal. clavula, Cal. raja* and *Cal. sukumum*) are currently not known. In Tanzania, amphistome cercariae of unidentified *Calicophoron* species were recorded in *Bul. forskalii* (Lotfy et al. 2010). Lotfy et al. (2010) suggested that besides *Cal. phillerouxi*, which is known from *Bul. forskalii*, two other *Calicophoron* species known in Tanzania, *Cal. bothriophoron* and *Cal. sukumum* with unknown snail hosts, cannot be ruled out. In the East African region, *Bul. abyssinicus* is reported as the intermediate host of *Cal. clavula* in Somalia (Sobrero 1962). Dinnik and Hammond (1968) suggested *Bul. globosus* as a likely snail host of *Cal. raja* as it is experimentally proven to be susceptible to infection. Four *Carmyerius* species with presently unknown snail hosts are *Car. bubalis, Car. dollfusi, Car. gregarious* and *Car. spatiosus*. Amphistome cercariae belonging to an unidentified species of the family Gastrothylacidae were recorded from *Cer. natalensis* in Kenya (Lotfy et al. 2010). The genus *Carmyerius* is one of four genera belonging to the family Gastrothylacidae. Hence, besides *Car. Mancupatus* and *Car. exoporus* already known from this snail host, one other *Carmyerius* species known in Kenya, *Car. spatiosus* with unknown snail hosts, cannot be ruled out (Lotfy et al. 2010). Besides *Cer. natalensis*, Wright et al. (1979) also suggested *Bul. forskalii* as likely snail hosts of *Car. spatiosus* in Zambia. To date, all four *Cotylophoron* species have unknown snail hosts. The other species with unknown snail hosts are *Bil. papillogenitalis, Cho. onotragi, Gas. crumenifer, Gig. symmeri* and *Ste. compactus*. However, even though not yet confirmed, *Bul. forskalii* has been speculated to act as the intermediate host of *Ste. compactus* (Dinnik 1965).

## Epidemiological features of amphistome infections in ruminants in East and Southern Africa

The epidemiology and prevalence of amphistomosis depend on several factors. These include the species of definitive and intermediate hosts (Rolfe et al. 1991), the potential of the flukes to infect these hosts (Dinnik 1964a; Dinnik & Dinnik 1954; Horak 1967), the topography and biological potential of the snail hosts (Dinnik 1964a; Horak 1971; Rolfe et al. 1991; Swart & Reinecke 1962a, 1962b), the definitive hosts’ management systems and their grazing habits as well as climate (Rolfe et al. 1991).

Data on amphistome infection prevalence are scarce for the reviewed countries and are currently only available from six countries (Table 2). The prevalence data are based on coprology and fluke counts with most studies having conducted in cattle. Because of difficulties in amphistome species identification, specific species prevalence data are lacking. Prevalence studies are limited for goats, sheep and wild ruminants. The available data show a high prevalence in the Kafue lechwe, but low prevalence rate in goats and sheep. In cattle, the coprological prevalence varies from 23.7% to 86.5%, while it varies from 25.5% to 96% on fluke counts, the high prevalence perhaps being explained by the fact that amphistome infection in ruminants is commonly because of several species. In the highlands of Kenya, *Cal. microbothrium, Cal. daubneyi* and *Cal. jacksoni* were recovered from a single animal and in few cases *Cal. sukari, Car. exoporus* and *Car. mancupatus* were present as well (Dinnik 1964a). Another combination of six species (*Cal. microbothrium, Cal. phillerouxi, Cal. raja, Cot. cotylophorum, Car. parvipapillatus* and *Ste. compactus*) was found in an ox in Zambia (Dinnik 1964a). Amphistomes recovered in slaughtered cattle were a combination of *Cal. microbothrium* and *Cot. jacksoni* in Tanzania (Keyyu et al. 2006). Infections with different amphistome species are also reported in Zimbabwe in cattle (Dube et al. 2004; Dube & Tizauone 2014) and in sheep and goats (Dube, Masanganise & Dube 2010). In addition, most amphistome species (85%) are shared between domestic and wild ruminant hosts (Table 1), providing a potentially stable source of infection among the ruminant animals. Furthermore, the availability of a wide range of the snail hosts (Table 1) with high biological potential also increases the successful propagation of amphistomes in the environment leading to increased infection exposure in ruminants. Limited routine anthelmintic treatment, particularly in rural communities who practice communal grazing, and the lack of effective drugs against amphistomes are also possible explanations for the high prevalence in domestic ruminants. An increase in the prevalence of amphistome infections has been reported in Western Europe (Foster et al. 2008; Mage et al. 2002; Murphy et al. 2008; Toolan et al. 2015). Besides an improvement in quality of diagnosis, the increase has also been attributed to the absence of an effective anthelmintic against amphistome infections (Mage et al. 2002).

**TABLE 2:** Prevalence of amphistomes in ruminants in east and southern African countries based on faecal egg and fluke counts.

Host	Location	Total examined	Positive	Prevalence (%)	95% CI	Publication year	References
**Faecal egg counts**							
Cattle	Tanzania	450	283	62.9	58.2–67.3	2015	Nzalawahe et al. 2015
Cattle	Tanzania	241	90	37.3	31.3–43.8	2014	Nzalawahe et al. 2014
Cattle	Uganda	233	158	67.8	61.3–73.7	2011	Howell 2011
Cattle	Kenya	344	108	31.4	26.6–36.6	2010	Kanyari et al. 2010
Cattle	Zambia	50	38	76.0	61.5–86.5	2008	Yabe et al. 2008
Cattle	Zambia	268	96	35.8	30.1–41.9	2007	Phiri et al. 2007a
Cattle	Zambia	101	33	32.7	23.9–42.8	2007	Phiri et al. 2007b
Cattle	Tanzania	482	302	62.7	58.2–67.0	2006	Keyyu et al. 2006
Cattle	Zambia	709	366	51.6	47.9–55.4	2006	Phiri et al. 2006
Cattle	Tanzania	301	168	55.8	50.0–61.5	2005	Keyyu et al. 2005
Cattle	Zimbabwe	16 264	4790	29.5	28.8–30.2	2005	Pfukenyi et al. 2005a
Cattle	Zimbabwe	12 472	6697	53.7	52.8–54.6	1999	Vassilev 1999
Cattle	Zimbabwe	796	490	61.6	58.1–64.9	1994	Vassilev 1994
Cattle	Kenya	1878	481	25.6	23.7–27.7	1993	Waruiru et al. 1993
	**Total**	**34 589**	**14 100**	**40.8**	**40.3–41.3**	**-**	**-**
Goats	Kenya	33	4	12.1	4.0–29.1	2009	Kanyari et al. 2009
Sheep	Kenya	54	16	29.6	18.4–43.8	2009	Kanyari et al. 2009
Buffalo	Uganda	10	6	60.0	27.4–86.3	2011	Howell 2011
Kafue lechwe	Zambia	22	11	50.0	28.8–71.2	2002	Kock et al. 2002
**Fluke counts**							
Cattle	Uganda	32	27	84.4	66.5–94.1	2011	Howell 2011
Cattle	Zambia	50	48	96.0	85.1–99.3	2008	Yabe et al. 2008;
Cattle	Zimbabwe	3225	822	25.5	24.0–27.0	2004	Dube et al. 2004
Cattle	Zimbabwe	1377	429	31.2	28.7–33.7	2002	Dube et al. 2002
	**Total**	**4684**	**1326**	**28.3**	**27.0–29.6**	**-**	**-**
Goats	Zimbabwe	3000	60	2.0	1.5–2.6	2010	Dube et al. 2010
Sheep	Zimbabwe	1000	60	6.0	4.7–7.7	2010	Dube et al. 2010
Kafue lechwe	Zambia	8	7	87.5	46.7–99.3	2012	Munang’andu et al. 2012
Wildebeest	Zambia	6	4	66.7	24.1–94.0	2012	Munang’andu et al. 2012
Kafue lechwe	Zambia	65	65	100.0	93.1–99.9	2011	Phiri et al. 2011
Kafue lechwe	Zambia	40	40	100.0	89.1–99.8	2010	Munyeme et al. 2010
Impala	South Africa	46	41	89.1	75.6–95.9	1983	Anderson 1983

Studies on animal-breed predisposition to amphistome infection are limited. Indigenous cattle breeds were observed to have a significantly higher prevalence and intensity than the exotic breeds and crosses in Kenya and Uganda (Howell 2011; Kanyari, Kagira & Mhoma 2010). In Tanzania, the Maasai Zebu cattle had a significantly higher prevalence than the Iringa Red cattle; however, the numbers of animals involved were too small for any meaningful interpretations to be made (Nzalawahe et al. 2015). Literature reports a variable effect of sex in domestic ruminants. Despite females tending to record higher prevalences than males, the associations were not significant (Kanyari et al. 2009, 2010; Keyyu et al. 2006; Phiri, Chota & Phiri 2007a; Phiri, Phiri & Monrad 2006). However, Pfukenyi et al. (2005a) observed significantly higher prevalences in pregnant and lactating cows compared with bulls, oxen and dry cows. Similarly, Howell (2011) reported a significantly higher prevalence in female cattle compared with males. The differences between sexes could probably be related to grazing patterns, sex hormones and treatment regimes.

Adult domestic ruminants are reported to have a significantly higher prevalence compared with young animals (Howell 2011; Kanyari et al. 2009, 2010; Keyyu et al. 2005, 2006; Nzalawahe et al. 2014; Pfukenyi et al. 2005a; Phiri et al. 2007a; Vassilev 1999). This is attributed to a long exposure time in adults leading to immunity against the pathogenic effects of immature amphistomes but still having the mature ones maintaining their high egg production capacity (Horak 1971). The resistance to amphistome re-infection in cattle was demonstrated clinically (Horak 1967) with no simultaneous studies on the cellular effector systems that characterise the acquired resistance. Mavenyengwa et al. (2008) showed that the resistance to *Cal. microbothrium* re-infection in cattle involves eosinophils and mast cells that are targeted at immature flukes. Epidemiologically, adults act as a constant source of infection, but clinical amphistomosis remains a problem for young animals, with adults grazing the same pastures exhibiting no clinical effects despite continued egg production (Boray 1959, 1969; Butler & Yeoman 1962; Rolfe et al. 1991). Amphistomes may survive for up to two years in the definitive hosts, providing a virtually constant source of infection for successive generations of snail hosts. In rural areas, the grazing management of young and adult animals may differ, where young animals graze around farms or homesteads while adults are trekked long distances to valleys, flood plains or swampy areas where they are exposed to high metacercariae-contaminated pastures (Keyyu et al. 2005, 2006).

Animal grazing area and/or habitat is significantly associated with prevalence and intensity of amphistomes in domestic ruminants (Howell 2011; Kanyari et al. 2009, 2010; Keyyu et al. 2005, 2006; Nzalawahe et al. 2014, 2015; Pfukenyi et al. 2005a; Phiri et al. 2006). The prevalence is highest in animals grazing in areas characterised by wetlands or swampy or marshy grazing areas where the distribution of suitable snail habitats is widespread. For instance, the highveld region in Zimbabwe, characterised by wet/swampy grazing areas where distribution of snail habitats is widespread, is associated with a higher prevalence compared with the lowveld which is characterised by dry land grazing with a focal distribution of snail habitats (Pfukenyi et al. 2005a, 2005b). Similarly, the presence of wetlands and high livestock density in the cattle grazing areas of the Zambian western and southern provinces is associated with an increased risk of acquiring amphistome infections (Phiri et al. 2006) and the same observations have been reported in Kenya (Kanyari et al. 2009, 2010), Tanzania (Keyyu et al. 2005, 2006; Nzalawahe et al. 2015) and Uganda (Howell 2011). In Tanzania, traditional communal grazing areas exhibited the highest prevalence of amphistomes compared with other sectors and this is attributed to heavy contamination of the habitats with eggs where intermediate host snails breed, because of high stocking densities with subsequent heavy metacercarial density on vegetation grazed by the animals especially during the dry season (Keyyu et al. 2005, 2006). Villages practising irrigation of crops are associated with high amphistome infection rates in Tanzania (Nzalawahe et al. 2014) as this provides favourable ecological conditions for growth of snail hosts and development of trematode larval stages.

The prevalence in domestic ruminants as measured by coprology follows a seasonal pattern with an increase towards the end of the dry season and a peak during the wet months of the year (Keyyu et al. 2005; Pfukenyi et al. 2005a; Phiri et al. 2007a; Reinecke 1983; Vatta & Krecek 2002). Outbreaks of acute clinical amphistomosis because of immature flukes are usually confined to the drier months of the year (Boray 1969; Butler & Yeoman 1962; Dinnik 1964a; Horak 1967, 1971; Rolfe et al. 1991; Vassilev 1999). Towards the end of the rainy season and onset of the dry season, conditions in permanent water sources become favourable for an increase in the number of the snail intermediate hosts, reaching their peak during the mid-to-end of the dry season (Chingwena et al. 2002a; Pfukenyi et al. 2005a, 2005b; Phiri et al. 2007b). As the snail hosts are extremely adaptable and prolific breeders, this ensures their widespread availability as well as heavy shedding of cercariae which encyst on vegetation surrounding the habitats (Dinnik 1964a). The proportion of infected snails increases from the end of the rainy season into the dry season (Chingwena et al. 2002a; Pfukenyi et al. 2005a). A combination of high snail numbers, asexual multiplication of the fluke in infected snails and survival of snails in suitable environments for several months may result in shedding of large numbers of cercariae. During this period, the infective metacercariae are spread over pastures surrounding permanent water sources where they can survive for several months. This coincides with pasture areas being narrowed around permanent water sources or wetland environments where animal concentration becomes high (Pfukenyi et al. 2005b). Contamination rates of these areas are increased, resulting in more snails being infected and high numbers of metacercariae on surrounding herbage, leading in turn to acute infections of animals with amphistomes. Thus, a build-up of immature flukes occurs, accounting for clinical amphistomosis outbreaks and low amphistome prevalence as measured by coprology. The outbreaks are common in ruminants that graze in marshy or swampy areas and are usually confined to the dry season. However, on irrigated pastures, moisture is often adequate for the survival of snail hosts and metacercariae, and hence outbreaks can occur throughout the year. In Tanzania, some villages practising year-round zero-grazing had high levels of amphistome infections attributed to the acquisition of cattle fodder from irrigation canals and swamps contaminated with metacercariae (Nzalawahe et al. 2014).

Development of amphistomes into adults takes 5–9 months (Dinnik & Dinnik 1962) and the prepatent period is 56–89 days (Dinnik & Dinnik 1962; Horak 1971). Five to 9 months after infection, the immature flukes become fully mature and this would lead to high faecal egg production and thus, account for the high prevalence during the rainy season as measured by coprology. During this period, abundant grazing and alternative water sources are available. Hence, drinking from and grazing around infected permanent water sources is greatly reduced. Furthermore, snail habitats and pastures are constantly flooded, and thus snails and the parasitic free-living stages are regularly flushed (Pfukenyi et al. 2005b). In summary, the intermediate and definitive hosts acquire most of the infection during the beginning and/or middle of the dry season. This results in immature fluke infections and clinical amphistomosis during the dry season and patent (mature fluke) infections during the wet months and at the end of the dry season. However, the timing may vary depending on location, length of the rainy season and the grazing habits of the animals (Pfukenyi et al. 2005b).

## Impact on production

Adult flukes are not associated with clinical amphistomosis (Mavenyengwa, Mukaratirwa & Monrad 2010). However, in heavy infections they have been hypothesised to cause weakness, recurrent ruminal tympany, ruminal atony, weight loss, anaemia and production losses (Anuracpreeda, Wanichanon & Sobhon 2008). They are also reported to be associated with inflammation of the mucosa and mucoid diarrhoea (Rolfe & Boray 1993). Based on coprology, poor body condition is reported to be significantly associated with high amphistome prevalence in cattle (Kanyari et al. 2010). A similar observation was noted in small ruminants (Kanyari et al. 2009), but the association was not significant. Cattle infected with more than 500 adult amphistomes had a significant reduction in final carcass mass when compared with controls (Marchand 1984; Dube & Tizauone 2014). The concurrent infection of amphistomes with other parasites known to depress growth rate such as strongyles (Kanyari et al. 2010), *Fasciola* species (Kanyari et al. 2010; Keyyu et al. 2006; Nzalawahe et al. 2014; Phiri et al. 2006; Yabe et al. 2008) and *Moniezia* species (Kanyari et al. 2010) is a likely explanation of the significant association between poor body condition and amphistome infections. However, further studies on the effect of adult amphistomes on production are required.

Clinical amphistomosis is caused by the immature flukes that lodge in the first 3 m of the small intestine (Mavenyengwa et al. 2010). The occurrence of clinical amphistomosis and subsequent clinical pathology in ruminants is dependent on the dose, pathogenicity of the species and the level of establishment of the metacercariae in the host’s small intestine (Horak 1967; Mavenyengwa et al. 2010). In ruminants, the disease is characterised by anorexia, anaemia, submandibular oedema, and hypoproteinemia, foul-smelling fetid diarrhoea, general weakness, polydipsia, and a reduction in feed conversion, weight and milk production and mortality in young animals (Boray 1969; Horak 1966; Mavenyengwa et al. 2010; Mohan 2011; Pillai & Alikutty 1995; Rolfe, Boray & Collins 1994; Spencer, Fraser & Chang 1996). Together with gastrointestinal nematodes, amphistome infection in cows can reduce milk production by approximately 0.4 L/day – 3 L/day (Mohan 2011; Spencer et al. 1996). Anthelmintic treatment of dairy cows infected with gastrointestinal nematodes (oxfendazole) and amphistomes (oxyclozanide) resulted in a significant increase in milk production, averaging 0.4 L/day (Spencer et al. 1996). The reduction in milk yield during clinical amphistomosis is associated with fetid diarrhoea (Mohan 2011). Mohan (2011) also reported anoestrus during clinical amphistomosis, while a functional obstruction or paralytic ileus of the intestine because of severe amphistomosis was reported in a cow (Yogeshpriya et al. 2011). Despite their ubiquity and abundance, as well as an increase in their prevalence in domestic ruminants, the economic importance of amphistome infections is not yet fully known and is likely to be underestimated in eastern and southern Africa – an area which requires further studies.

## Diagnosis

Diagnosis of amphistomes in live animals is still dependent on faecal detection of eggs (Rieu et al. 2007) and this method only detects the presence of adult rumen fluke infection (Malrait et al. 2015). The filtration technique with sieves and sedimentation is the most accurate method to identify eggs in faeces (Horak 1971). Using contrast stains such as methylene blue or methyl green to distinguish amphistome eggs from *Fasciola* ova is advisable. One drawback of this diagnostic method is that, in acute infections, it is highly probable not to find eggs or only very few as this is usually associated with massive infection with immature flukes (Horak 1971). The agreement between a modified McMaster method and necroscopic diagnosis of amphistome infection is reported to be high (Rieu et al. 2007) with no significant differences being observed between the two methods. The modified McMaster method showed a significant association between eggs per gram (epg) counts and parasite burden; more than 100 epg indicated the presence of more than 100 adult amphistomes in the rumen and/or reticulum (Rieu et al. 2007). Similarly, the mini-FLOTAC is a reliable method of assessing the presence of adult amphistome infection with both sensitivity and specificity being above 0.9 (Malrait et al. 2015). A good correlation was found between faecal egg count (FEC) and estimated rumen fluke burden with a FEC > 200, indicating the presence of more than 200 adult rumen flukes in the rumen and/or reticulum (Malrait et al. 2015). The adult worms are difficult to identify to species level because most have thick robust bodies in which the internal organs are difficult to see. Even by using histological techniques, species identification is still problematic (Lotfy et al. 2010). As the flukes responsible for disease are sexually immature, specific identification is made even more difficult and the diagnosis has to rely on the dubious procedure of identifying a few adult worms, which may be present in the rumen of the animal (Horak 1971). Because of these problems, PCR-based techniques providing rDNA ITS2 sequences have proven to be reliable tools to identify amphistome species and to determine their phylogenetic relationships (Itagaki et al. 2003; Rinaldi et al. 2005). Using cercariae and rediae from snail hosts and adult flukes obtained from slaughterhouses, Lotfy et al. (2010) confirmed ITS2 as a good molecular marker for amphistome identification that can also be used to determine phylogenetic and amphistome–snail associations.

The clinical diagnosis of amphistomosis remains challenging as immunological techniques are usually not conclusive (Horak 1967, 1971). Croposcopic examination cannot be used for the early diagnosis of clinical amphistomosis which is vital for prompt treatment before considerable damages and economic losses are incurred. For the identification of immature flukes, the recommended method is to mix approximately 10 g of faeces with 100 mL – 200 mL of water (Horak 1971). The mixture is allowed to stand for 5 min, followed by decanting any supernatant fluid and then repeating the procedure four to five times. Young flukes, resembling small white or pink rice grains, will be seen after pouring the sediment on a black surface for examination (Horak 1971). In dead animals, postmortem, pathological and clinical pathological findings combined with the presence of immature flukes in the affected intestines would be confirmative. The gross pathological, histopathological and clinical pathological findings are as described in the literature (Horak 1966, 1967, 1971; Horak & Clarke 1963; Mavenyengwa et al. 2005, 2008, 2010; Pillai & Alikutty 1995). An indirect ELISA performed to detect coproantigens in faecal supernatants of 100 cattle known to be infected with *Gas. crumenifer* had a sensitivity of 74% (Kandasamy & Devada 2011). Generally, the sensitivity of the indirect ELISA ranges from 74% to 86% and its specificity from 79% to 90% (Hassan et al. 2005; Kandasamy & Devada 2011; Salib et al. 2015; Sanchis et al. 2012; Shivjot et al. 2009). However, Shivjot et al. (2009) reported a very low specificity of 23.7%. The ELISA was shown to be more specific and accurate but less sensitive than Western blotting for the diagnosis of amphistome infections in cattle and buffaloes (Salib et al. 2015). Results indicate the feasibility of ELISA for the detection of coproantigens of amphistome infections, especially for the diagnosis of immature amphistomosis where faecal examination may not reveal eggs.

## Control

The available epidemiological information on amphistomes of ruminants in the area under review can be used to design appropriate integrated control measures. Options available for the control of amphistome infections are mainly based on chemical treatment, non-chemical management practices and immunological control.

### Chemical treatment

Chemical control involves treatment with a product that is effective against both adult and immature flukes. Oxyclozanide given twice, three days apart, has a high efficacy against both adult and juvenile amphistomes (Rolfe & Boray 1987) and a high anthelmintic performance in cattle (Arias et al. 2013; Rolfe & Boray 1987; Spencer et al. 1996) and small ruminants (Paraud et al. 2009; Rolfe & Boray 1988; Sanabria et al. 2014). Studies in Tanzania showed a reduced efficacy of levamisole–oxyclozanide combination against amphistomes in cattle (Keyyu et al. 2008) and this is of great concern as they are the commonly available drugs in the country. However, levamisole is widely used to treat nematode infections in livestock and it is not intended as treatment against trematodes. When given orally at a higher dosage (10 mg/kg), closantel has a high efficacy against mature flukes (Arias et al. 2013). However, treatment of mature flukes with intra-ruminally (Rolfe & Boray 1993) or subcutaneously administered (Malrait et al. 2015) closantel is not effective. In countries where oxyclozanide is unavailable, the use of closantel to treat against mature flukes is recommended. When administered at high doses (50 mg/kg and 100 mg/kg), niclosamide has 94% – 99% efficacy against immature amphistomes (Rolfe & Boray 1987).

Even though it is not of direct benefit to the animal, treatment against mature amphistomes will prevent egg laying and thus reduce pasture contamination (Horak 1971), while treatment against the immature flukes will reduce the impact of the disease. During the rainy season, mature amphistomes are expected and anthelmintic treatment with drugs effective against adult flukes is indicated. The strategic anthelmintic treatment against mature amphistomes should be given in adult animals at the end of the rainy season (Pfukenyi et al. 2005a, 2005b) or beginning of the dry season (Keyyu et al. 2005) to reduce the opportunity for snail infections. The timing of this treatment is dependent on local factors, length of the rainy season and the grazing habits of the animals. Where possible, adult animals targeted for treatment should have high levels of infection based on coprology. Depending on availability, oxyclozanide or closantel can be administered during this period to treat against mature amphistomes.

Disease epidemiology indicates that large burdens of immature amphistomes are expected during the dry season. As adult animals are resistant to the pathogenic effects of the migrating immature amphistomes, the target for treatment would be young animals being exposed to the infection for the first time (Pfukenyi et al. 2005a). Hence, the first anthelmintic treatment can be administered in young animals during the mid-dry season period when maximum migration of immature amphistomes starting 3–4 weeks after infection in the early dry season is expected. To remove potentially high burdens of immature amphistomes acquired later in the dry season, a second treatment could be given towards the end of the dry season (Pfukenyi et al. 2005a). Oxyclozanide or niclosamide can be administered during this period to treat against immature amphistomes. In communal areas, animals are communally grazed and for optimum benefits, the recommended anthelmintic treatments should be well organised and preferably done at the same time within a village. Where cattle are dipped for the control of ticks, dip tank facilities where all animals are gathered during dipping sessions could be used for organised fluke control (Pfukenyi et al. 2005a, 2005b).

The efficacy of medicinal plant extracts against amphistomes has recently been evaluated. The ethanol extract of *Punica granatum* L. (Lythraceae), commonly known as pomegranate, is highly effective against amphistomes in naturally infected sheep (Lalhmingchhuanmawii, Veerakumari & Raman 2014). The authors concluded that the plant extract could be successfully used as an anthelmintic to treat amphistomes in domestic ruminants. Similarly, an aqueous extract of *Acacia concinna* (Willd.) DC. (Fabaceae) significantly reduced egg counts of amphistomes in naturally infected sheep and also restored the haemato-biochemical profile to normal in extract-treated sheep (Priya, Veerakumari & Raman 2013). However, efficacy of the *P. granatum* and *A. concinna* extracts was not established in immature amphistomes. Other studies have also shown medicinal plants extracts to be effective against amphistomes (Elango & Rahuman 2011; Kamaraj et al. 2010).

Chemical control of the snail hosts through application of molluscicides such as niclosamide may also be done. To achieve cost-effective control, this type of control should be done during the peak transmission period to reduce numbers of infected snails and cercarial shedding. Thus, the application could be done during the mid-dry and towards the end of the dry season (Pfukenyi et al. 2005b). The application is practical and economical in areas where snail habitats are focal and not widespread, but regular application may be necessary because of the rapid recovery of the snail populations during brief periods of favourable conditions. However, molluscicide application causes environmental pollution and also kills non-targeted aquatic organisms (Roberts & Suhardono 1996).

### Immunological control

Hafeez and Rao (1981) showed that the lifespan and pathogenicity of amphistomes developing from gamma irradiated (2 or 3 krad) metacercariae were greatly reduced with the higher irradiation dose resulting in the complete absence of the flukes in infected animals. Single vaccination of kids and lambs with 3000 irradiated (2 or 3 krad) metacercariae stimulated a significant degree of resistance against challenge and the resistance was more pronounced in the group vaccinated with a higher irradiation dose (Hafeez & Rao 1981). Earlier, Horak (1967) successfully immunied sheep, goats and cattle against massive artificial infections with *Cal. microbothrium*. The animals were given immunising infections with at least 40 000 metacercariae and later challenged with larger doses of metacercariae (Horak 1967). Cattle were the most suitable subjects for immunisation with the immunity being effective for at least a year post-immunisation (Horak 1967, 1971). Mavenyengwa et al. (2008) demonstrated that cattle acquire resistance to amphistome infection. This resistance is targeted at immature amphistomes and it involves eosinophils and mast cells. However, despite promising immunisation results, the mass production of snail hosts and metacercariae remains a challenge and a major limiting factor (Horak 1967, 1971; Mavenyengwa et al. 2006; Swart & Reinecke 1962a, 1962b). Thus, the success of a large-scale immunisation program is dependent on a viable metacercariae mass production system.

### Non-chemical control

The best preventive method against amphistome infections is to keep domestic ruminants from infected pastures (Pfukenyi et al. 2005b). Fencing-off or drainage of wetlands or marshy/swampy areas and provision of clean pastures and cercariae-free water in troughs are advised (Roberts & Suhardono 1996). Similarly, habitat management through vegetation clearance is also effective in controlling the snails (Woolhouse & Chandiwana 1990). However, habitat management and complete separation of stock from snail habitats are only practical and economical where the snail habitats are focal and not widespread (Pfukenyi et al. 2005b). These control methods are not feasible in communal grazing areas. It is also important to repair any leaks in dams and water troughs as they can create an ideal habitat for the snail hosts.

## Conclusions

Twenty-six amphistome species belonging to nine genera from three families occur in domestic and wild ruminants in the area under review and seven snail species belonging to four genera from two families act as their intermediate hosts. Eighty-five per cent of the amphistome species are shared between domestic and wild ruminant hosts. Some snails are intermediate hosts of amphistome species belonging to the same genus or to different genera – a phenomenon not yet fully elucidated. Only nine (34.6%) of the amphistome species have known snail intermediate hosts, while most (65.4%) have unknown hosts. The epidemiology of amphistomosis depends on the species of definitive and intermediate hosts and the potential of the flukes to infect these hosts, the topography and biological potential of the snail hosts, the management systems of the definitive host and their grazing habits and climatic factors. Based on current epidemiological information, the strategic anthelmintic treatment against mature amphistomes should be given in adult animals at the end of the rainy or early dry season. The anthelmintic treatment in young animals against immature amphistomes should be administered during the mid-dry and towards the end of the dry season. Further research is necessary to determine the economic importance of amphistomosis, amphistome–snail associations, efficacy of different anthelmintics and to develop diagnostic tests that can detect prepatent infections in the definitive host.
